# Batch Sedimentation Studies for Freshwater Green Alga *Scenedesmus abundans* Using Combination of Flocculants

**DOI:** 10.3389/fchem.2017.00037

**Published:** 2017-06-19

**Authors:** Raghu K. Moorthy, M. Premalatha, Muthu Arumugam

**Affiliations:** ^1^Department of Energy and Environment, National Institute of Technology, TiruchirappalliTiruchirappalli, India; ^2^Microbial Process and Technology Division, National Institute of Interdisciplinary Science and Technology (CSIR)Trivandrum, India; ^3^Academy of Scientific and Innovative Research, Council of Scientific and Industrial Research (CSIR)New Delhi, India

**Keywords:** algae, biomass harvesting, sedimentation, flocculation, efficiency

## Abstract

Microalga is the only feedstock that has the theoretical potential to completely replace the energy requirements derived from fossil fuels. However, commercialization of this potential source for fuel applications is hampered due to many technical challenges with harvesting of biomass being the most energy intensive process among them. The fresh water microalgal species, *Scenedesmus abundans*, has been widely recognized as a potential feedstock for production of biodiesel (Mandotra et al., 2014). The present work deals with sedimentation of algal biomass using extracted chitosan and natural bentonite clay powder as flocculant. The effect of flocculant combination and different factors such as temperature, pH, and concentration of algal biomass on sedimentation rates has been analyzed. A high flocculation efficiency of 76.22 ± 7.81% was obtained at an algal biomass concentration of 1 ± 0.05 g/L for a settling time of 1 h at 50 ± 5°C with a settling velocity of 103.2 ± 0.6 cm/h and a maximum surface conductivity of 2,260 ± 2 μS/cm using an optimal design in response surface methodology (RSM). Biopolymer flocculant such as chitosan exhibited better adsorption property along with bentonite clay powder that reduced the settling time significantly.

## Introduction

Harvesting of algal biomass is primarily a solid-liquid operation in a top-down approach from algal growth to downstream applications. The unit operation accounts for nearly 30% of total production cost along with a dewatering procedure to enhance the availability of concentrated algal biomass for the next stage (Schlesinger et al., 2012). The important factors that affect any harvesting technique include (a) nature of algal species (b) age of algal culture volume and (c) harvesting time. The choice of a harvesting technology has been influenced by many other factors such as growth medium, end product requirement, the energy content of the biomass and energy costs thereby involved (Milledge and Heaven, 2013). Output with lower moisture content from harvesting techniques such as sedimentation-flocculation, filtration, and flotation require a dewatering step such as a secondary technique to reduce moisture content further. Examples of dewatering include unit operations such as drying or low-speed centrifugation for a reduced volume (Schlesinger et al., 2012). Subsequently, downstream processing techniques can be performed to obtain various value-added products. Among them, bio-fuel applications are the most popular one as there is a wider scope for their implementation in existing petroleum refineries. The extraction of algal oil (not as in extracted phase) can be used as a blend in existing petroleum products to enhance fuel efficiency and reduce production costs (Mandotra et al., 2014).

There are numerous microalgal species isolated from different water sources with higher oil (25–77% of dry matter), protein (43–71% of dry matter) and carbohydrate (10–30% of dry matter) content (Al Hattab et al., 2015). Freshwater species such as *Scenedesmus abundans* in particular has been recently reported to be a potential raw material for high-quality biodiesel production. It is known to survive even at harsh environmental conditions such as alkaline pH without much damage to its cells. Modified CHU-13 medium under moderate temperature conditions was identified as the favorable growth medium for microalgal species *S. abundans* with sufficient nitrogen stress provided by a commonly found fertilizer component like potassium nitrate for high growth rate (Mandotra et al., 2014). Higher lipid content was reported for the microalgal biomass at nearly 43% (in terms of % of dry cell weight; Mandotra et al., 2014).

Sedimentation involves settling of solid particles in liquid suspensions mainly due to gravity. To achieve high separation efficiency, the combined effect of gravitational forces and density difference between algal biomass and liquid is required for dilute algal biomass suspensions. Shape, size, and composition of aggregates or flocs formed may be diverse according to the type of microalgal species considered (Salim et al., 2012). Algal biomass harvesting can be carried out using sedimentation-flocculation techniques in an energy efficient manner. Compared to energy-intensive centrifugation for large volume, these techniques, in general, can bring down the energy requirements by a factor of three in terms of kWh m^−3^ (Salim et al., 2012; Milledge and Heaven, 2013). Recently, numerous process approaches using sedimentation-flocculation principles have been analyzed (Table 1). Density difference can be induced in a solid-liquid suspension either by changing the pH of the suspension (Liu et al., 2013) or through addition of chemical flocculants such as alum or iron oxide (Wang et al., 2014) in the media suspensions. Introduction of bio-flocculants like *Chlorella vulgaris* CNW11 extracted from cell wall of *C. vulgaris* JSC-7 into the suspensions was also shown to lead to the formation of aggregates or flocs (Alam et al., 2014).

**Table 1:** Summary of different sedimentation-flocculation techniques for algal biomass harvesting.

**Microalgal species**	**Nature of flocculation**	**Flocculants used**	**Flocculation Efficiency (%)**	**References**
*Scenedesmus* sp.	Flocculation induced by pH	Induced by pH decrease to 4.0	90	Liu et al., 2013
Mix of green algae	Coagulation-flocculation	Ecotan and Tanfloc (tanin-based)	95	Gutierrez et al., 2015
*Chlorella* sp.	Coagulation-flocculation	Chitosan	99.40	Ahmad et al., 2011
*Chlorella vulgaris*	Bio-flocculation	Seed powder of clearing nut, *Strychos potatonum*	99.68	Razack et al., 2015
*Chlorella vulgaris* JSC-7	Freely suspended microalgae (or bio-flocculation)	*C. vulgaris* CNW11 and *S. obliquus* (at 1:2 ratio each)	85 per hour	Alam et al., 2014
*Neochloris oleoabundans*	Bio-flocculation	*Tetraselmis suecica* in ratio of 0.25	Nearly 60	Salim et al., 2012
*B. braunii, C. ellipsoidea*	Magnetic flocculation	Cationic polyacrylamide iron-oxide	More than 95	Wang et al., 2014

In a previous study, usage of cationic polyacrylamide-iron oxide (CPAM-Fe_3_O_4_) particles as a magnetic flocculant for settling of algal cells (*B. braunii* and *C. ellipsoidea*) within 10 min was reported. A per cent recovery of more than 95% was achieved through electrostatic attraction and bridging mechanisms. It used a neodymium-based permanent magnet (0.02 T of magnetizing intensity) that resulted in a reduction of settling time by 50% in the absence of the CPAM polymer indicating the ineffectiveness of the technique. It also stated that charge neutralization could be considered as a separation mechanism only at lower pH-values (Wang et al., 2014). Another study suggested the use of an electrocoagulation-flocculation technique involving sacrificial aluminum (Al) or iron (Fe) anodes for algal cells of *Chlorella* sp. to attract solid particles on the basis of difference in surface charge. The main disadvantage encountered was in the use of electrodes that were highly prone to fouling, resulting in a reduction in flocculation efficiency (Milledge and Heaven, 2013).

Inorganic flocculants can be toxic and reduce the presence of microalgae in the growth media possibly due to chemical interactions. Use of cationic polyelectrolyte (dosage of 2–25 mg/L) was reported to attain were of higher biomass concentration for flocculating freshwater microalgae (Milledge and Heaven, 2013). For example, chitosan is a crystalline biopolymer derived from skeletons of crustaceans such as marine shrimps. It is biocompatible, anti-bacterial, eco-friendly, and highly soluble in aqueous acetic acid solution (Li et al., 2013). Typical applications where chitosan was in use include water treatment (Ahmad et al., 2005), dietary supplement, biomedical devices, microcapsule implants for controlled release in drug delivery, photographic papers (Sigma-Aldrich Inc., 2015).

Natural bentonite clay is essentially composed of silica and alumina. Use of bentonite clay required maintaining a pH range of 5–6 in order to promote aggregate formation due to its highly inherent, negative charges (Ahmad et al., 2005). The lower economic costs and availability of bentonite from clay mines suggest a reduction in usage of chitosan for flocculation of algal cells. This could possibly result in the wide use of natural bentonite clay as a flocculant for algal biomass harvesting.

For estimation of process parameters, a statistical tool was required to build a design of experiments at a certain number of levels and analyze its responses. The minimum and maximum limits for these parameters were identified from previous literature or preliminary studies in order to determine the effect of varying factors on the response parameters and provide an optimized condition (Design-Expert Software, Version 9.0.6.2, 2015). For example, the use of central composite design (CCD) as response-surface model was reported for process optimization of bio-flocculation as the first attempt using response surface methodology (RSM; Razack et al., 2015). Mathematical modeling could be performed from the three-dimensional response surface according to best-fit design model. Literature reported the accurate use of RSM to find the optimum flocculation efficiency for the separation of algal cells with magnetic chitosan and proposed mathematical models for the same (Goudarzi et al., 2015). In the present work, a process relying on gravity settling, with a change in pH and addition of flocculant combination of chitosan and bentonite clay, has been considered for the microalgal species *S. abundans*. Dynamic process parameters affecting the sedimentation-flocculation such as the concentration of algal biomass, temperature, pH, and concentration of flocculants were considered for optimization using RSM.

## Experimental

### Microalgal growth

*S. abundans* (NCIM 2897) was obtained from the National Collection of Industrial Microorganisms at National Chemical Laboratory (Pune, India). To obtain higher biomass productivity, literature suggested the use of modified CHU-13 media for the microalgal species at optimized concentration that was prepared as described (Mandotra et al., 2014). For comparison of growth kinetics, microalgal species were grown in another culture medium (Algae Broth Composition, refer to Supplementary Material Table A1). A 400 ml of algal culture was grown in 1,000 ml Erlenmeyer flask, sealed with cotton and maintained at a temperature of 25 ± 2°C and a light intensity of 2,000 lux under 16/8 h light/dark cycle. The flasks were gently shaken two times a day, and no air or carbondioxide was supplemented during the experimental period to determine the nature of growth kinetics. Five percent of the mother culture was inoculated into the medium for each algal culture. Every 3 days, culture samples were withdrawn aseptically and growth measurements were taken using UV-Visible Spectrophotometer (Spectroquant Pharo 300, Merck) at 680 nm. Corresponding cell number was found out by direct microscopic cell count using a Brightline Heamocytometer (Neubauer counting chamber, depth of 0.1 mm) and an optical microscope (Nikon Eclipse TS100 Model) and was expressed in terms of 10^4^ cells/ml. The 40 ml samples taken in pre-weighed centrifuge tubes were centrifuged at 10,000 rpm for 5 min, oven-dried overnight at 60°C and reweighed. The dry biomass yield was then expressed in g/L. For experiments related to harvesting of microalgal cells by sedimentation-flocculation technique, 15 L algal culture supplied with an air flow rate of 7 L/min (or at an aeration rate of 0.4 L of air per 1 L of algal culture) under a light intensity of 7,000 lux (or ~140 μmol m^−2^s^−1^) was grown utilizing tap water source with 30% salinity. Algal biomass was harvested at the start of the stationary phase. Splitting up of coenobium structure of microalgal species into single cells was observed for this algal culture due to higher aeration rate maintained during these experiments (Vasumathi et al., 2013).

### Materials and preparation of solutions

Shrimp (or sea) shell samples were collected from coastal areas in Nagercoil district, Tamil Nadu, India. Natural bentonite clay powder was obtained from the clay mines in Taluka-Mandvi, Kutch, Gujarat, India. 1 N sodium hydroxide (NaOH) and hydrochloric acid (HCl) solutions were prepared and used for adjustment of pH during sedimentation-flocculation experiments. Thirty-seven percent of HCl solution was diluted with low conductivity water to obtain 4% HCl solution for use in purification of chitosan. All analytical grade chemicals or reagents unless stated otherwise were purchased from Merck and HiMedia Laboratories (India).

### Purification of chitosan

Chitosan obtained after purification was to be utilized as a flocculant in the settling process of algal biomass. Extraction of chitosan from shrimp (or sea) shells was performed using simple acidic treatment and alkaline hydrolysis methods (Islam et al., 2011). Acid treatment of shrimp or sea shells was done by using 4% hydrochloric acid [1:14 (w/v)] at room temperature for 36 h. Alkaline treatment with 5% (w/v) sodium hydroxide for solid to solvent ratio of [1:12 (w/v)] of acidified shrimp (or sea) shells was done at a temperature of 75°C for 24 h in order to form chitin. Alkaline hydrolysis of the residue was carried out for deacetylation of chitin to form chitosan using 50% sodium hydroxide [1:14 (w/v)] at room temperature for 72 h. A powdered form of the organic end product containing chitosan as a major constituent was obtained at the end (mortar & pestle were used for crushing purpose). To confirm the presence of chitosan in the prepared sample, FTIR spectroscopy analysis was also performed.

### Density analysis

A clean, dry empty specific gravity bottle of 25 ml (with a stopper) was weighed (W_1_). To estimate the density of the dry solid sample, it was transferred to the bottle and weighed (W_2_). The solid sample was soaked in about 10 ml low conductivity water for 2 h. Again, the bottle was filled with water and the weight of the bottle and its contents was determined (W_3_). The bottle was emptied and cleaned before being completely filled with water, and its weight was noted down (W_4_). Then, at room temperature conditions, the bulk density was calculated as follows:

(1) $$\begin{array}{l} Bulk\;density\;(\text{at }30\pm 5{}^{\circ}\text{C})=\{({\text{W}}_{2}-{\text{W}}_{1})/[({\text{W}}_{4}-{\text{W}}_{1})\\ \;-({\text{W}}_{3}-{\text{W}}_{2})]\}\times 1,000{\text{kg/m}}^{3} \end{array}$$

### Lipid content estimation

The most common method to estimate lipid content in microalgal biomass is through Folch extraction method (Mandotra et al., 2014; Anand and Arumugam, 2015). In this method, 50 mg of dry algal biomass powder is added to mixture of chloroform and methanol in 2:1 (v/v) ratio. Samples were uniformly mixed at room temperature for 15–20 min after which the mixture was centrifuged at 7,000 rpm (room temperature) for 7 min to separate the cell debris from the supernatant. Collection of the supernatant and subsequent washing with 0.9% (w/v) sodium chloride solution in 1:4 (v/v) ratio were done. The mixture was subjected to vortex for few seconds and centrifuged at 3,000 rpm for 5 min to get two separated layers. The upper phase was removed, and lower chloroform phase containing lipid was kept for slow evaporation and drying overnight at room temperature. Pre-weighed bottles were used to determine lipid content (as % of dry cell weight) and its productivity was calculated as:

(2) $$\begin{array}{l} \text{Lipid productivity }(\text{g}.{\text{L}}^{-1}{\text{day}}^{-1})\\ =\text{biomass productivity }(\text{g}.{\text{L}}^{-1}{\text{day}}^{-1})\times \text{lipid content }(\%) \end{array}$$

### Experimental replication and statistical analysis

Experiments studying microalgal growth involved the use of triplicates. Density analysis was performed in duplicates for flocculant samples considered at varying room temperature conditions (in a range of 30 ± 5°C). Estimation of the lipid content and batch sedimentation tests were carried out in duplicates. Fixed error analysis was done throughout the study and reported. For batch sedimentation studies as per experimental design laid out by RSM, the overall standard deviation was calculated and reported for each of the response parameters.

### Design of experiments

As a part of an experimental design by RSM, an optimal (custom) design was chosen to determine the effect of four dynamic process variables namely, the concentration of algal biomass, the surrounding temperature, pH, and concentration of flocculant over the two responses, flocculation efficiency (% per unit settling time) and settling velocity. Three different levels (at minimum, median, and maximum values) were used for this analysis. The response surface design model is Optimal (Custom) Design. Its features include suitability for processes where greater control of design variable is required and includes irregular regions in the domain. The number of variable parameters input can be equal to or more than four, suitable constraints can be added to obtain optimality criteria and higher order models for process optimization can be formulated with minimal error. Twenty-five experimental trials along with five replicates were considered in total (Design-Expert Software, Version 9.0.6.2, 2015).

### Batch sedimentation tests

Sedimentation-flocculation tests were carried out in 500 ml graduated glass measuring cylinders with a 3.2 cm diameter and a fixed depth of 24 cm filled with algal culture. Extracted chitosan and natural bentonite clay powder were added as flocculant at a combination of 1:1. Blank sedimentation test for algal biomass sample (without the addition of flocculants and devoid of any change in process parameters) was performed to determine the effect of flocculant combination and pH. Adjustment of pH was made by drop-wise addition of 0.1 N NaOH or HCl solution. Hot temperature condition (50 ± 5°C) and cold temperature condition (15 ± 5°C) were maintained using a water bath (supplied by Rays Scientific Instruments, Chennai, India) and reusable flexible chiller ice packs (supplied by Easy Ice, Indotech, Mumbai, India) respectively. Effect of the light intensity was not considered in these experiments because control of its variation in real-time was difficult (associated with broad daylight). Thus, a light intensity of up to 100 lux (light conditions at the laboratory) was allowed for all trials except one that was exposed to sunlight in real-time inside a specially constructed algal cultivation shed (at the Department of Energy & Environment, National Institute of Technology Tiruchirappalli, India). Samples were drawn from the top surface (within a depth of 2 cm from the top) at defined time intervals and from the bottom surface (at a depth of 24 cm from top) at end of each batch sedimentation test and analyzed using an optical microscope (Nikon Eclipse TS100) and the images were captured at a 40X magnification. A sample of 3 ml was drawn from the top surface during the settling process for determination of cell count and optical density at 680 nm. Corresponding height of hazy interface was noted down throughout the settling time. The settling process was allowed till no change in optical density (for flocculant compatibility trials) and cell count (for RSM experimental trials), of the sample was obtained. All trials were conducted with duplicates under observation. For compatibility trials, batch sedimentation tests were performed with six different trials and data collected for further analysis.

(3) Flocculation efficiency (%)=[1−(A/B)]×100

(4) $$\begin{array}{l} \text{Flocculation efficiency per cent per hour }(\%\;h^{-1})\\ =\text{Flocculation efficiency/t} \end{array}$$

where A stands for final cell number or optical density for the algal solution at the end of settling process and B for initial cell number or optical density for the algal solution at the start of settling process, and finally t is the time taken for attaining constant cell number or optical density at the top surface level of the algal solution.

At fixed concentration of algal biomass, temperature and pH, assume an ideal case of flocculation efficiency of 100% during the compatibility studies. Then,

(5) $$\begin{array}{l} \text{Total settling time }(\text{in hours})\\ =100/(\text{Flocculation efficiency per hour}) \end{array}$$

The effect of the variation of concentration of algal biomass, temperature, initial pH and concentration of flocculant (combination of chitosan and bentonite clay) on settling of the microalgal cells were carried out using RSM to obtain optimum process parameters with an objective of achieving maximum flocculation efficiency and settling velocity.

### Analytical methods

Optical density was measured using UV-Visible Spectrophotometer (Spectroquant Pharo 300, Merck, Darmstadt, Germany) at 680 nm. Values of pH were found using a calibrated pH-009(I) Digital pH meter (Surat, India). Surface conductivity was measured using conductivity meter (SenseION 5, Hach, Germany). Fourier Transform-Infrared Spectroscopy (FTIR) analysis was performed with FTIR Spectrometer (Perkin Elmer/Spectrum 2, Massachusetts, USA). Elemental analysis for dry algal biomass (in powdered form) collected at the initial and final instance of settling process was performed using CHNS/O Analyzer (Perkin Elmer/2400 Series II, Massachusetts, USA) in sulfur mode.

## Results and discussion

### Kinetics of microalgal growth

**Figure 1:**
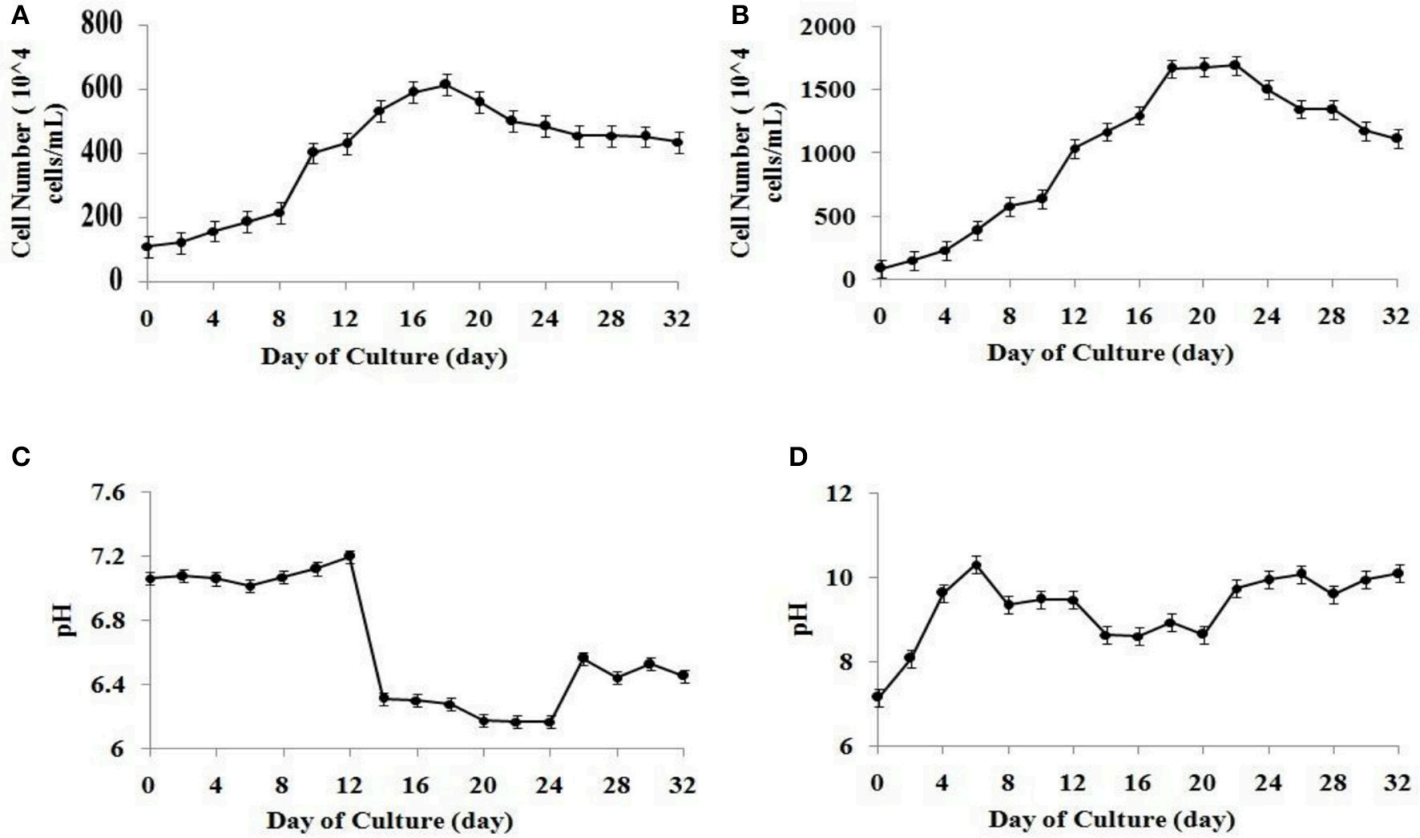
Growth curve for *S. abundans* based on cell number in **(A)** algae broth composition **(B)** modified CHU-13 medium. Variation of pH during growth phase in **(C)** algae broth composition **(D)** modified CHU-13 medium.

The algal cell concentration of microalgal species *S. abundans* followed the well-known growth kinetics in the form of a sigmoidal curve. The exponential growth phase was observed between the fourth and twentieth day of culture in the modified CHU-13 medium. A slight decrease in the amount of active cells was noted after 22 days of culture, possibly due to insufficient nutrient availability (Figures 1A,B). Considering the cell number N_o_ (at time t_o_ = 4) and N_t_ (at time t_t_ = 20) observed between fourth and twentieth day of culture, the specific growth rate (SGR) can be calculated as:

(6) $$\text{SGR}=[\text{ln }({\text{N}}_{\text{t}}{\text{/N}}_{\text{o}})]/({\text{t}}_{\text{t}}-{\text{t}}_{\text{o}})$$

(7) Doubling=SGR/(ln 2)

(8) Doubling time=1/Doubling

It may be noted that the cell density was significantly higher in the modified CHU-13 medium which infers higher growth rate when compared to algae broth composition (common media). Using the above microalgal growth data in modified CHU-13 medium, calibration curves between cell number and optical density as well as between dry biomass yield and optical density were plotted with an accuracy of more than 90%. The maximum dry biomass yield and biomass productivity obtained at the start of stationary phase for *S. abundans* in modified CHU-13 medium were 1.450 ± 0.112 g L^−1^ and 0.066 ± 0.022 g L^−1^day^−1^ respectively.

### Effect of media pH on microalgal growth

The variation of the media's pH during the microalgal growth was analyzed for the validation of the selection of a suitable growth media. A higher growth pH of 9.4 ± 0.1 was observed for modified CHU-13 medium from start of stationary phase when compared to similar state of growth for algal broth composition. It was found that alkaline pH had to be maintained during algal harvesting for highly efficient flocculation process. Since it was observed that pH of algal culture varied in the range of high alkalinity (between 9 and 10) during the stationary phase of growth (Figures 1C,D), modified CHU-13 medium was selected for further experiments.

### Characterization of algal biomass samples

Lipid content (in per cent of dry cell weight) was estimated using the Folch extraction method. The lower solvent phase, containing lipid, was collected and dryed. Replicate sample was also considered for the experiment to reduce errors. A higher lipid content of 52 ± 2% (in per cent of dry cell weight) was obtained with a lipid productivity of 0.034 ± 0.001 g/L.day. The latter validates the results given in previous reports claiming that microalgal species *S. abundans* is a potential feedstock for biofuel applications (Mandotra et al., 2014). An elemental analysis of dry algal biomass powder was performed a CHNS/O Analyzer with an accuracy of <0.3%. Dry *S. abundans* biomass (sample weighing 1.557 mg) was placed in an elemental furnace, combusted in pure oxygen environment (sulfur mode) at 975°C. From Table 2, the carbon-to-nitrogen ratio was calculated and found to be 10.361, which lie in the range required (10–30) for the use of biomass in biogas applications such as reported in the earlier literature (Skorupskaite et al., 2015).

**Table 2:** CHNS Analysis of dry algal biomass powder (*S. abundans*) with an accuracy of <0.3%.

**Element**	**Composition (weight %)**
Carbon	44.76
Hydrogen	5.07
Nitrogen	4.32
Sulfur	0.48

**Figure 2:**
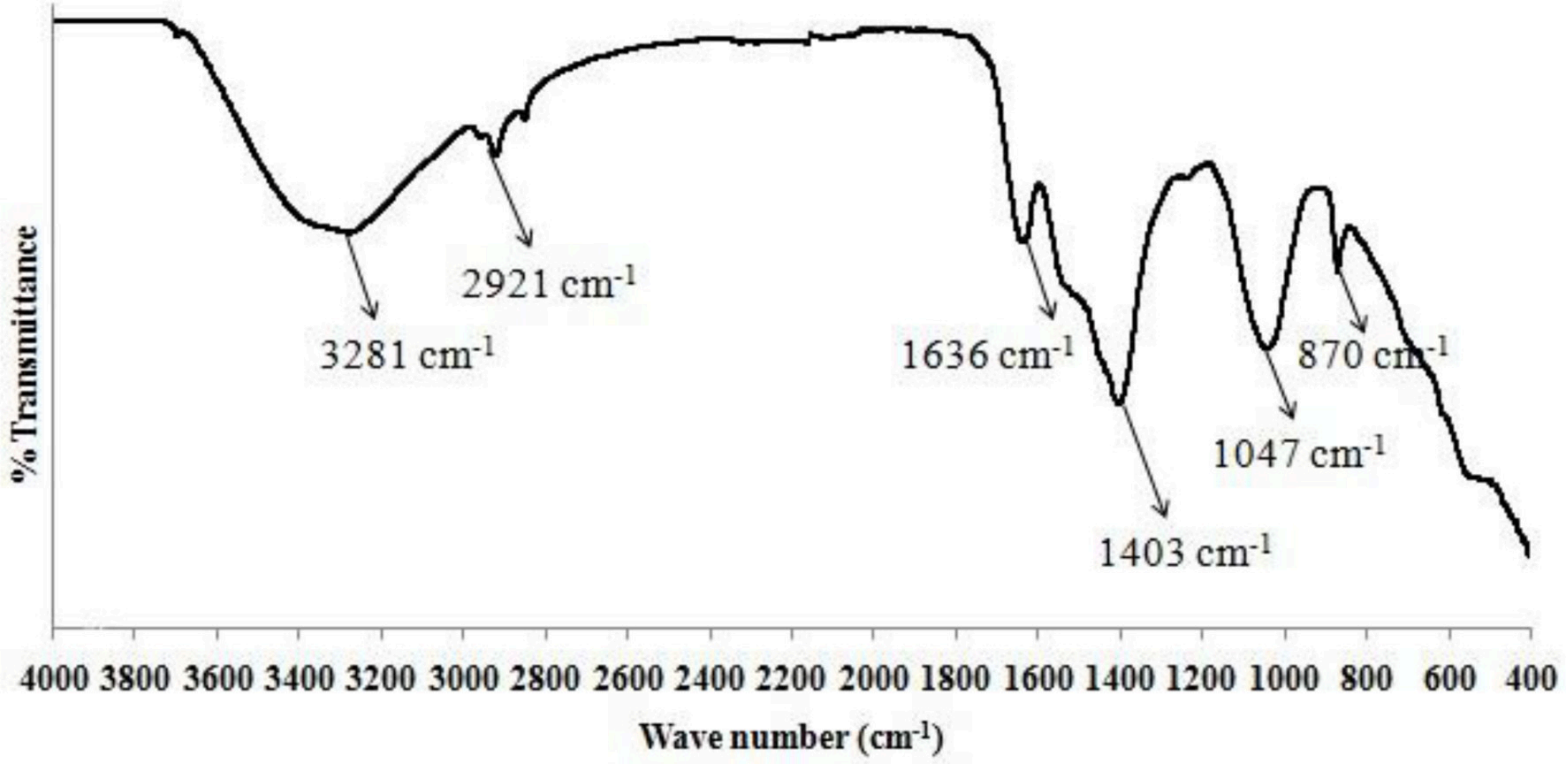
FTIR analysis of sediment sample of dry algal biomass (Credits: Perkin Elmer Instrument).

The sediment sample of dry algal biomass was collected from bottom of the cylindrical vessel (at a depth of 24 cm), both before start of the settling process as well as after 1 h of settling time. The sedimentation process was carried out at room temperature conditions. To confirm the presence of functional groups, a qualitative analysis using FTIR Spectroscopy was performed in the in the wave number range of 4,000–400 cm^−1^ (Ajayan et al., 2015). The results (Figure 2) conformed to the peak values obtained for both the samples of dry algal biomass with a deviation of <5%.

### Effect of pH and conductivity on algal biomass samples with addition of flocculant combination

**Figure 3:**
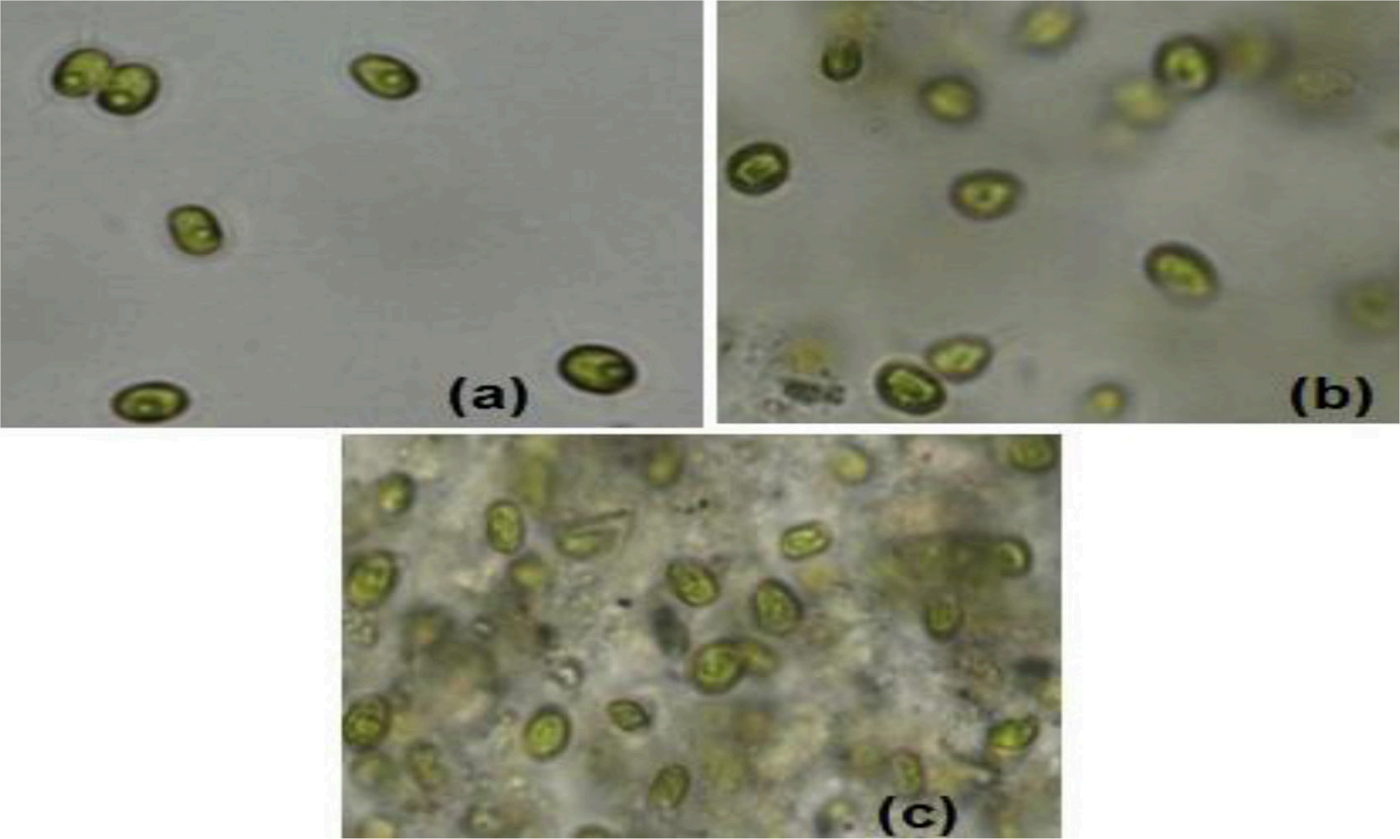
Microscopic images (at 40X magnification) after settling process: **(a)** normal algal biomass sample with no flocculants **(b)** algal biomass from separate upper layer when combination of flocculants is used **(c)** algal biomass from mixed bottom layer when combination of flocculants is used (Credits: Nikon Eclipse TS100).

Sample experiments were conducted to study the variation of pH and conductivity for flocculants in medium with or without the presence of algal biomass. For all samples involved, 100 ml of medium (use of tap water or water sample of very low conductivity) was utilized and flocculants used were extracted chitosan and natural bentonite clay powder. It was observed that pH does not vary in the presence of only bentonite clay powder in medium (no algal biomass present). But, in the case of extracted chitosan as well as in the presence of algal biomass in medium, significant pH variation was found to occur (Table 3). Both pH and conductivity of the algal biomass sample increased significantly on the addition of chitosan and bentonite clay in the medium, which indicated that there was a profound effect if flocculants are combined in an optimum ratio. Microscopic images of above samples considered as in Table 3 were taken using an optical microscope (Nikon Eclipse TS100) to compare the appearance of flocculants when mixed with algal biomass (Figure 3). It shows an aggregation of algal cells indicating that there was a profound effect of added flocculants on the settling process. It is possibly due to the attractive forces that develop between the oppositely charged algal cells and chitosan. The pull exerted by negatively charged bentonite clay may have led the denser combination of algal cells (chemically unaffected) and chitosan to sediment down toward the bottom eventually.

**Table 3:** Variation of pH and conductivity for different flocculants in medium with or without algal biomass.

**Sample**	**pH ± 0.1**	**Maximum Conductivity ± 2 (μS/cm)**
Medium only	8.2	167
Medium + 0.55 g/L algal biomass	9.1	198
Medium + 0.15 g/L chitosan	9.4	170
Medium + 0.15 g/L bentonite	8.3	153.4
Medium + 0.15 g/L chitosan + 0.15 g/L bentonite	9.5	175.3
Medium + 0.55 g/L algal biomass + 0.15 g/L chitosan	9.4	235
Medium + 0.55 g/L algal biomass + 0.15 g/L bentonite	9.1	206
Medium + 0.55 g/L algal biomass + 0.15 g/L chitosan + 0.15 g/L bentonite	9.8	226

### Flocculant compatibility studies

The analysis of the difference in density among the algal biomass and set of flocculants was performed using the gravimetric method. The true density of the dry algal biomass (in powdered form) was found to be 1,237 kg/m^3^ and indicating algal biomass can auto-flocculate in the liquid medium to a certain extent, but settling time extends beyond 8 h. It was calculated as follows:

(9) $$\begin{array}{l} \text{True density}=(\text{Bulk density})\\ \;\times \;[1-(\text{True porosity }(\text{in }\%)/100)] \end{array}$$

Thus, settling kinetics has to be improved by a change in process conditions or use of flocculants. Similarly, true density of flocculants (in powdered form) was found out as given in Table 4. On comparison with the density of water, the relative difference in density of flocculants clearly indicates that it is one of the major driving forces in sedimentation-flocculation technique and reduces settling time significantly.

**Table 4:** Density analysis of flocculants.

**Material**	**Density (in kg/m^3^ at 30 ± 5°C)**
Extracted chitosan	1651.152
Natural bentonite clay	1581
Raw sea shell powder	1642.152

For all experiments in the flocculant compatibility studies, the process parameters such as the concentration of algal biomass, temperature, and pH were kept constant, and so, the sedimentation-flocculation was affected simply by the presence of flocculants or their combination alone (Table 5). The samples for analysis at each time interval were taken from the top surface level of the suspended solution. The algal cells were stained using 1% Evans' blue solution to check the number of viable cells (Liu et al., 2013). Since the values for cell viability during all the trials ranged between 93 and 97%, the algal cells were considered to be not affected by either the addition of flocculant nor changes in surrounding environment conditions in the form of temperature, pH, and light intensity.

**Table 5:** Process conditions considered for flocculant compatibility studies.

Concentration of algal biomass (all trials)	0.550 ± 0.05 g/L
Concentration of flocculant used	0.15 ± 0.001 g/L
Initial pH-value (all trials)	9.4 ± 0.4
Temperature (at laboratory)	33.5 ± 0.4°C
Sedimentation depth (all trials)	24 cm

The settling velocity for each sample was found out as a slope of the plot between height under consideration and settling time (use of tangent method). For the said purpose, a free, open source graphing software was utilized (Graph Software, Version 4.4.2). A flocculation efficiency of 78.60% (per 12 h) based on optical density was obtained when the algal biomass solution (*S. abundans*) of concentration of 0.55 g/L was subjected to direct addition of a combination of flocculants namely, extracted chitosan and natural bentonite clay powder, each of concentration of 0.15 g/L (Table 6, refer to Supplementary Material Figures 1.1 and 1.2). Although highest flocculation efficiency calculated was 81.12% for the sample of algal biomass and bentonite clay powder, the surface charge in terms of electrical conductivity was observed to be lower, and thus, the addition of chitosan becomes important for overall settling kinetics in terms of attaining higher settling velocity and required alkaline pH in the medium. The settling velocity varies rapidly in the first 90 min of settling process and lowers down for the rest of the time under observation. It reached a maximum of 0.258 ± 0.01 cm/min in the case of algal biomass sample with the addition of extracted chitosan and natural bentonite clay powder as flocculant combination (refer to Supplementary Material Tables 1.1–1.4). Corresponding results for the surface charge in terms of electrical conductivity indicate that the settling process can be made rapid due to the presence of extracted chitosan with maximum electrical conductivity observed for the same.

**Table 6:** Comparison chart of all trials involved in flocculant compatibility studies.

**Sample**	**Total settling time ± 1.6 (h)**	**Settling velocity ± 0.6 (cm/h)**	**Flocculation efficiency ± 7.81 (% efficiency per 12 h)**	**Maximum electrical conductivity ± 2 (μS/cm)**
Algal biomass without any flocculant	26.5	2.51	45.24	198
Algal biomass without any flocculant at higher temperature of 50 ± 5°C	35.1	6.02	34.20	237
Algal biomass without any flocculant at algal cultivation shed (for higher light intensity and temperature)	20.3	7.55	59.04	321
Algal biomass with chitosan	34.2	10.31	35.04	234
Algal biomass with bentonite	14.8	9.35	81.12	206
Algal biomass with bentonite and chitosan	15.3	10.84	78.60	226

### Experimental trials using response surface methodology (RSM)

**Figure 4:**
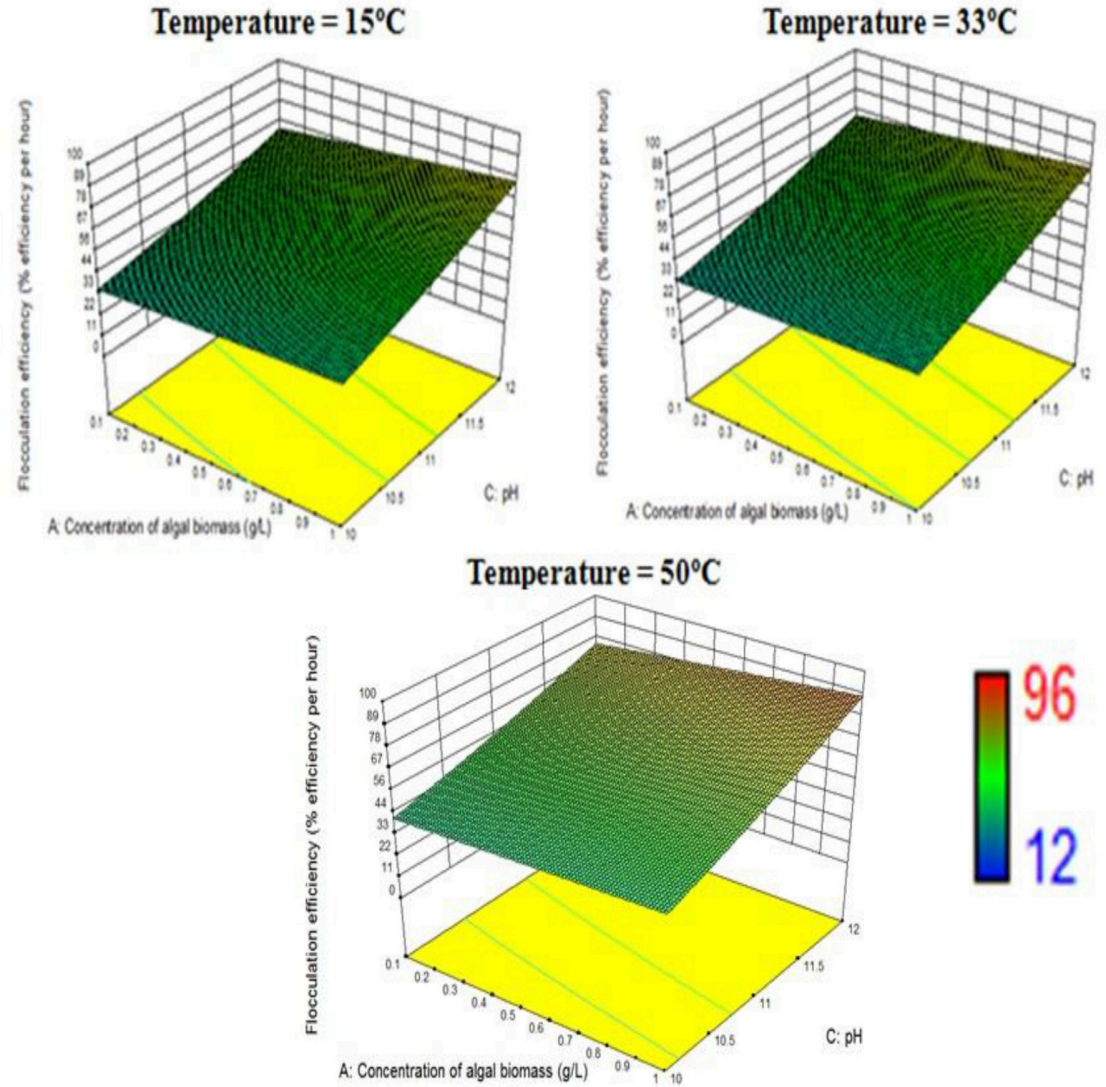
Effect of temperature on flocculation efficiency at a combined flocculant concentration of 0.005 g/L.

**Figure 5:**
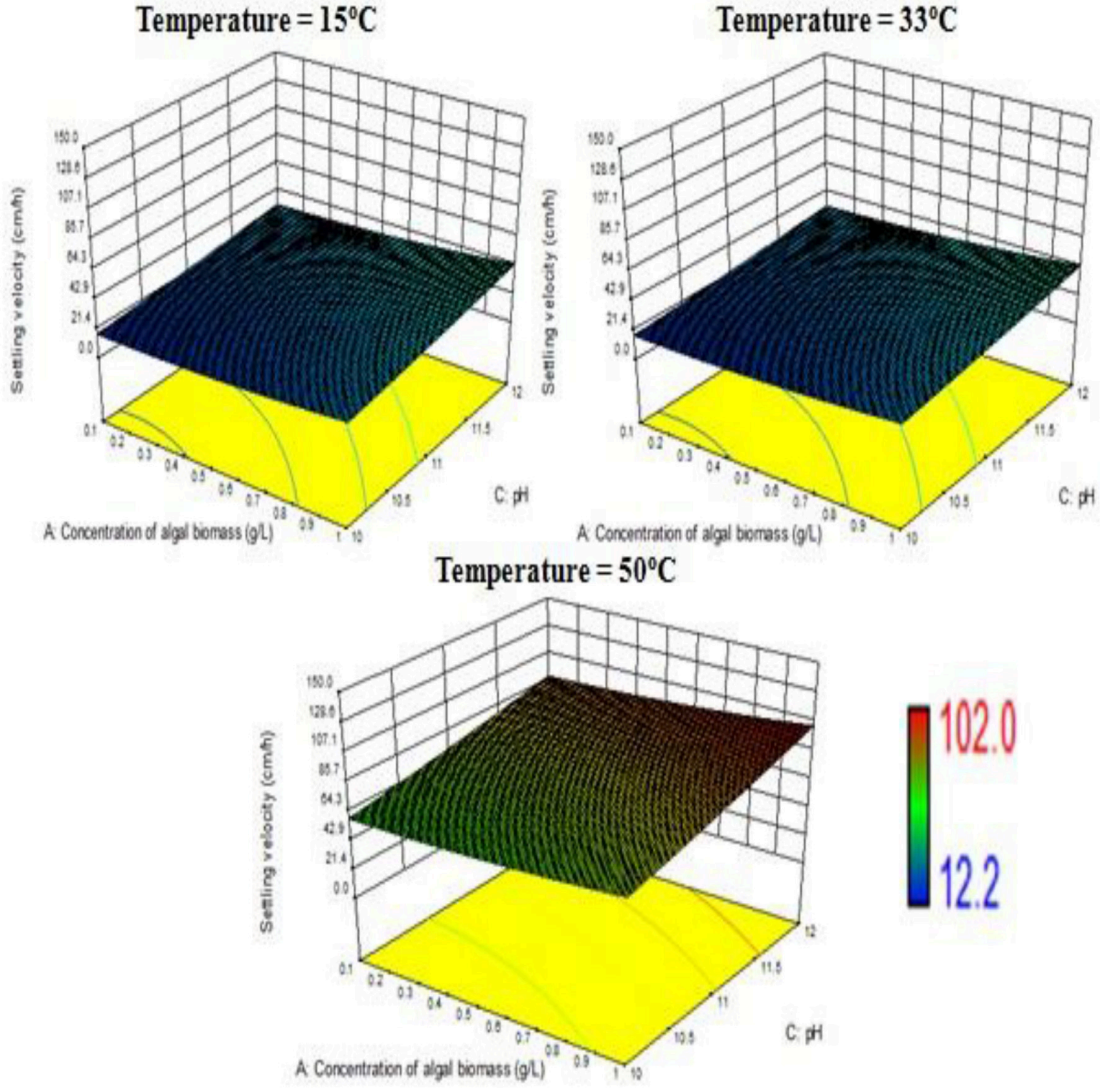
Effect of temperature on settling velocity at a combined flocculant concentration of 0.005 g/L.

The response surface design model is an Optimal (Custom) Design. Its features include suitability for processes where greater control of design variable is required and includes irregular regions in the domain, the number of variable parameters input can be equal to or more than four, and suitable constraints can be added to obtain optimality criteria, higher order models for process optimization can be formulated with minimal error. According to the minimum, median and maximum levels (3 levels) set for each of the four varying process parameters, namely concentration of algal biomass, surrounding temperature, initial pH and concentration of flocculant (1:1 ratio), a three-dimensional surface was generated on which flocculation efficiency and settling velocity were evaluated as responses (Table 7, refer to Supplementary Material Table A2). Based on previous literature, the response surface analysis was conducted over a limited range of lower concentration of flocculant combination (1:1 ratio) and alkaline pH (between 10 and 12; Ahmad et al., 2011; Li et al., 2013; Design-Expert Software, Version 9.0.6.2, 2015). Then, higher concentration of algal biomass of 1 g/L (maximum limit), lower concentration of flocculant combination of chitosan and bentonite clay (1:1 ratio) of 0.005 g/L each and alkaline pH of 12 at room temperature condition favored maximum flocculation efficiency of nearly 77% per hour of settling time (Figure 4). Since there was no significant increase in flocculation efficiency at a lower concentration of algal biomass at room temperature condition, the response surface in terms of velocity was analyzed at 0.55 and 1 g/L. Higher concentration of algal biomass of 1 g/L (maximum limit), a lower concentration of flocculant combination of chitosan and bentonite clay (1:1 ratio) of 0.005 g/L each and alkaline pH of 12 at room temperature condition favored a settling velocity of nearly 43 cm/h. Therefore, it has been found out that maximum flocculation efficiency was favored by a higher concentration of algal biomass of 1 g/L, a lower concentration of flocculant combination of chitosan and bentonite clay (1:1 ratio) of 0.005 g/L each and alkaline pH of 12 at room temperature of 33°C. With an increase in the surrounding temperature to 50°C, flocculation efficiency for the process increased to a maximum of 85%. Thus, there is a significant increase in flocculation efficiency at hot temperature condition with an increment of nearly 20% as compared to cold temperature condition (15°C). The response surface in terms of settling velocity at a fixed lower concentration of flocculant combination of chitosan and bentonite clay (1:1 ratio) of 0.005 g/L was analyzed at different temperature conditions (Figures 4, 5). There is a significant increase in settling velocity with the increase in temperature. It attained a maximum of nearly 104 cm/h at hot temperature condition (50°C) with a lower concentration of algal biomass of 1 g/L and alkaline pH of 12. Therefore, it has been found out that maximum flocculation efficiency and settling velocity can be achieved by higher concentration of algal biomass (1 g/L), a lower concentration of flocculant combination of chitosan and bentonite clay (0.005 g/L each) and alkaline pH 12, maintained at a temperature of 50°C.

**Table 7:** Limits for various process parameters in Response Surface Methodology (RSM).

Concentration of algal biomass	0.1 to 1 ± 0.05 g/L
Concentration of flocculant used	0.005 to 0.3 ± 0.001 g/L
Initial pH-value range	4.0 to 14.0 ± 0.1
Temperature	15 to 50 ± 5°C
Sedimentation depth	24 cm
Type of design model	Optimal (Custom) Design
Number of trials	25

**Figure 6:**
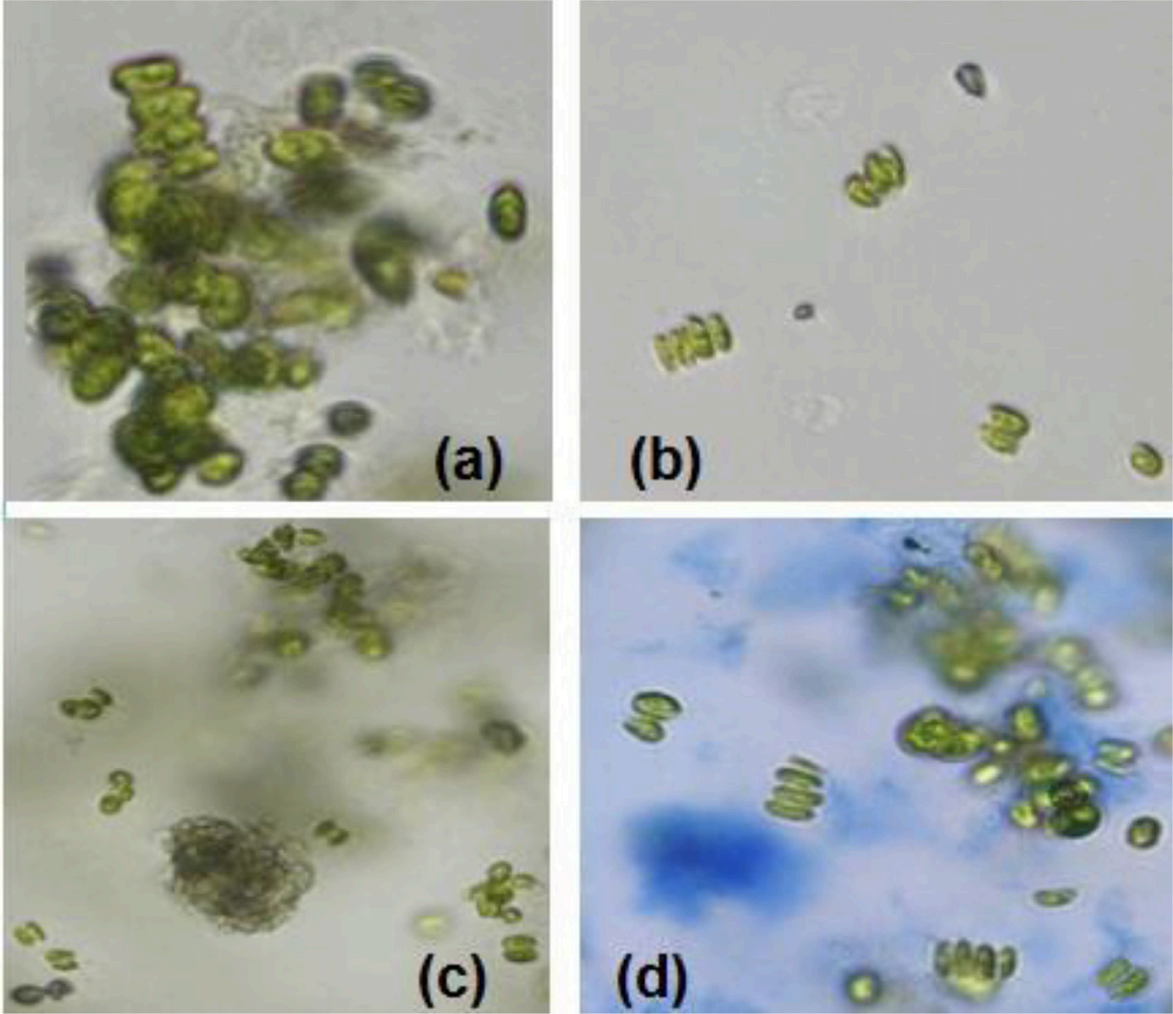
Microscopic images (at 40X magnification) after settling process with algal biomass at optimized conditions: **(a)** initial sample at top surface level (time *t* = 0) **(b)** final sample at top surface level (time *t* = settling time to attain constant cell density) **(c)** final sample at bottom surface (time *t* = settling time to attain constant cell density) **(d)** final sample (stained with 1% Evans' blue) at bottom surface level (time *t* = settling time to attain constant cell density; Credits: Nikon Eclipse TS100).

For the above results, the experiments involved the utilization of microalgal species *S. abundans* grown in the modified CHU-13 medium. The algal biomass was harvested at stationary phase, where maximum dry biomass yield obtained was 1.450 ± 0.482 g/L whose density was 1,237 kg/m^3^ through a sedimentation-flocculation technique. For a time of 1 h, the optimized process parameters were the concentration of algal biomass at 1 ± 0.05 g/L, a temperature of 50 ± 5°C, initial pH at 12 ± 0.1 and concentration of flocculant combination of chitosan and bentonite (1:1 ratio) at 0.005 ± 0.001 g/L. Thus, from the experimental run at the previously mentioned process condition, a flocculation efficiency of 76.42 ± 7.81% per hour was achieved with a settling velocity of 103.2 ± 0.6 cm/h. The microscopic images of the samples collected during the experimental run at optimized conditions showed an aggregation of algal cells before the start of settling process on the top surface of suspension under consideration and the same nature being replicated at bottom surface level (Figure 6). It indicated that addition of the flocculant combination affects the settling process.

### Flocculant sensitivity analysis at optimized conditions

In order to determine the effect of flocculant combination on the settling process, four different cases were considered: (a) algal biomass at optimized concentration and room temperature without any changes to pH and without any addition of flocculants (normal sample) (b) algal biomass at optimized concentration, temperature, pH, and without any addition of flocculants (to determine effect of pH) (c) algal biomass at optimized concentration, temperature, pH, and with addition of flocculant combination of chitosan and bentonite clay in 1:1 ratio (to determine effect of flocculant combination) and (d) algal biomass at optimized concentration, temperature, pH, and with addition of flocculant combination of chitosan and bentonite clay in 2:1 ratio respectively (to determine effect of change in concentration ratio for flocculant combination). In absence of flocculant, the flocculation efficiency was higher for the settling process on account of alkaline pH that induces instability in the suspension and imparts more amount of negative charge on the algal cells. It possibly led to the formation of unstable aggregates and thereby induced flocculation. In presence of flocculant addition in 1:1 concentration ratio between extracted chitosan and natural bentonite clay, the surface charge (in terms of conductivity) was the maximum (at 2,260 μS/cm) on the flocs or aggregates formed possibly due to adsorption of algal cells on to the larger surface area made available by fine particles of chitosan and bentonite clay. It induced a significant density difference between the aggregates and fluid coupled with instability conditions provided by the alkalinity of dilute algal suspension. It resulted in significantly higher flocculation efficiency of nearly 76.22% and settling velocity of 103.2 cm/h (refer to Supplementary Material Figures 1.3 and 1.4). When the concentration ratio of flocculant combination was increased to 2:1 between extracted chitosan and natural bentonite clay respectively with other process conditions at optimized level, there was no profound effect observed in the magnitude of flocculant efficiency or settling velocity. In comparison with the above cases (Table 8), it can be inferred that the sample at optimized process conditions provided maximum flocculation efficiency and settling velocity.

**Table 8:** Analysis of optimized conditions to determine effect of flocculant combination.

**Sample**	**Flocculation efficiency ±7.81 (% efficiency per hour)**	**Settling velocity ± 0.6 (cm/h)**	**Maximum electrical conductivity ± 2 (μS/cm)**
Algal biomass of 0.55 g/L without any flocculant or change in parameters	4.55	3.3	674
Algal biomass of 1 g/L at initial pH of 12 and higher temperature of 50 ± 5°C without any flocculant	46.36	96	1,875
Algal biomass of 1 g/L at initial pH of 12 and higher temperature of 50 ± 5°C with chitosan and bentonite of 0.005 g/L each	76.22	103.2	2,260
Algal biomass of 1 g/L at initial pH of 12 and higher temperature of 50 ± 5°C with 0.01 g/L chitosan and 0.005 g/L bentonite	63.79	96.6	1,832

### Mathematical models

Mathematical models for both flocculation efficiency and settling velocity were developed using ANOVA (statistical approach through analysis of variance) subject to some specific conditions: (a) No chemical interaction takes throughout the settling process (a) Laminar flow regime is assumed for the dilute algal suspension (b) No external forces act on flocculating particulate matter (c) The process parameters were considered as numeric factors (or values that can be directly observed or measured) (d) The minimum and maximum limits for different process parameters involved shall be treated as tight constraints and so, the model equation was considered as bounded. In the present study, it has been specifically validated only for *S. abundans* microalgal species with the use of modified CHU-13 nutrient medium.

A quadratic design model for flocculation efficiency was developed using ANOVA (statistical approach through analysis of variance) with a standard deviation of ±7.81%. For each of the model terms, an *F*-value was calculated to relate its variance with the residual variance. The Model *F*-value of 13.82 implied that the model was significant (refer to Supplementary Material Table A3). Probability values (*p*-value) of less than 0.05 associated with each of the *F*-value were found out and were considered to have a significant effect on the design model (Design-Expert Software, Version 9.0.6.2, 2015). In this case, C, D, AC, BC, CD, C^2^ were significant model terms, where A: concentration of algal biomass (g/L), B: temperature (°C), C: pH-value, D: concentration of flocculant (g/L). The reduced quadratic model for flocculation efficiency (% per hour) was given as:

(10) $$\begin{array}{l} \text{Flocculation efficiency}=130.908-36.108*A-1.780*B\\ \;-25.181*C+25.228*D+4.203\\ \;*A*C-14.468*C*D+0.016*B^{2}\\ \;+1.670*C^{2}+201.828*D^{2} \end{array}$$

A cubic design model for settling velocity was also developed with a standard deviation of ±0.6 cm/h. In this case, A, B, D, AB, AC, BC, BD, CD, A^2^, B^2^, C^2^, D^2^, B^2^C, BD^2^, CD^2^ were significant model terms (refer to Supplementary Material Table A4). The reduced cubic model for settling velocity (cm h^−1^) was given as:

(11) $$\begin{array}{l} \text{Settling velocity}=10.820-28.133*A+2.645*B-0.997\\ \;*C+600.570*D+0.140*A*B+1.910\\ \;*A*C-0.706*B*C-12.339*B*D\\ \;-43.603*C*D+13.136*A^{2}-0.046\\ \;*B^{2}+0.563*C^{2}-1836.123*D^{2}+0.012\\ \;*B^{2}*C+0.007*C^{2}*B+38.788*B*D^{2}\\ \;+130.362*C*D^{2} \end{array}$$

**Figure 7:**
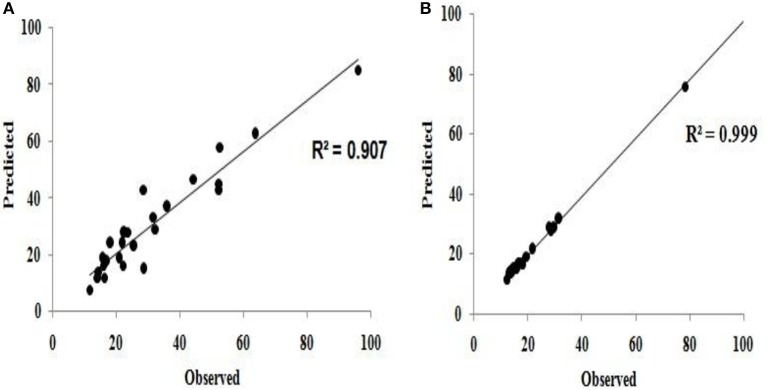
Comparison of predicted and observed (experimental) values for **(A)** flocculation efficiency **(B)** settling velocity.

The predicted values using the model equations (Equations 10, 11) and observed (or actual experimental) values of flocculation efficiency were compared and found to be qualitatively good (Figure 7).

## Conclusions

In this study, the effect of variation of the process parameters such as the concentration of algal biomass, temperature, initial pH, and concentration of flocculant (extracted chitosan and natural bentonite clay powder) on the batch settling of the microalgal cells was carried out. Settling velocity and flocculation efficiency were calculated as the response to the sedimentation-flocculation technique developed for algal biomass harvesting. Response surface methodology (RSM) was utilized to obtain optimum process parameters to achieve maximum flocculation efficiency and settling velocity. The concentration of algal biomass of 1 ± 0.05 g/L, a lower concentration of flocculant combination of chitosan and bentonite clay (1:1 ratio) of 0.005 ± 0.001 g/L each and alkaline pH of 12 ± 0.1 at temperature 50 ± 5°C was identified as the optimized condition. Batch sedimentation studies for the collection of concentrated algal biomass analyzed the settling kinetics and made possible identification of suitable process parameters including temperature and pH for further optimization. The determination of the effect of flocculants on settling kinetics led to the selection of a combination of flocculants as chitosan and bentonite clay at low dosage to maximize flocculation efficiency and settling velocity. Process optimization using RSM was performed and a maximum flocculation efficiency of 76.22 ± 7.81% per hour of settling time and maximum settling velocity of 103.2 ± 0.6 cm per hour of settling time was achieved. A reduced quadratic model and a reduced cubic model for prediction of flocculation efficiency and settling velocity respectively with an accuracy of above 90% based on batch settling kinetics were formulated subject to specific condition. It infers that the sedimentation-flocculation mode of algal biomass harvesting shall be achieved with high efficiency with the use of a flocculant combination of chitosan and bentonite clay possibly at lower production costs.

## Author contributions

RM conducted the experimental analysis of batch sedimentation tests for algal biomass using flocculant combination. MP conceived theoretical inputs on process parameters affecting batch sedimentation of algal biomass (chemical engineering aspects). MA initiated the research problem on collection of highly concentrated algal biomass through energy-efficient unit operations and gave significant scientific inputs on their effect on algal cells (microbiological aspects). All authors involved in the analysis of results obtained and manuscript preparation.

### Conflict of interest statement

The authors declare that the research was conducted in the absence of any commercial or financial relationships that could be construed as a potential conflict of interest. The reviewer IZB and handling Editor declared their shared affiliation, and the handling Editor states that the process nevertheless met the standards of a fair and objective review.
