# Supplementary material for: Batch Sedimentation Studies for Freshwater Green Alga Scenedesmus abundans Using Combination of Flocculants
Source: Front Chem. 2017 Jun 19;5:37. doi: 10.3389/fchem.2017.00037 (PMC5475385; doi:10.3389/fchem.2017.00037)
Supplement: Supplementary file 1 [file Table1.DOCX]

Supplementary Material

Batch sedimentation studies for freshwater green alga *Scenedesmus abundans* using combination of flocculants

Raghu Krishna Moorthy^1*^, M. Premalatha^1^, Muthu Arumugam^2^

^1^Department of Energy & Environment, National Institute of Technology, Tiruchirappalli, India

^2^Biotechnology Division, CSIR-National Institute of Interdisciplinary Science and Technology, Trivandrum, India

*** Correspondence:**Raghu Krishna Moorthy
raghukmoorthy@gmail.com

**1 TABLES**

Table 1.1: Preliminary experimental trial for algal biomass sample of concentration at 0.55 g L^-1^ (normal sample without any flocculant)

| **Sl. no.** | **Time (min)** | **Height (cm)** | **Settling velocity (cm/min)** | | **Cell number (10^4 cells/mL)** | | **Optical density (at 680 nm)** | | **Conductivity (µS/cm)** |  |  |
| --- | --- | --- | --- | --- | --- | --- | --- | --- | --- | --- | --- |
| 1 | 0 | 0 | - | | 757 | | 0.576 | | 196 |  |  |
| 2 | 30 | 3 | 0.100 | | 602 | | 0.458 | | 196 |  |  |
| 3 | 60 | 6 | 0.100 | | 594 | | 0.452 | | 196 |  |  |
| 4 | 90 | 7.5 | 0.083 | | 587 | | 0.447 | | 195.6 |  |  |
| 5 | 120 | 9.5 | 0.079 | | 540 | | 0.411 | | 196 |  |  |
| 6 | 150 | 10.2 | 0.068 | | 539 | | 0.41 | | 198 |  |  |
| 7 | 180 | 11.5 | 0.063 | | 536 | | 0.408 | | 198 |  |  |
| 8 | 210 | 12.3 | 0.058 | | 506 | | 0.385 | | 198 |  |  |
| 9 | 240 | 13 | 0.054 | | 466 | | 0.355 | | 199 |  |  |
| 10 | 270 | 14 | 0.051 | | 636 | | 0.484 | | 199 |  |  |
| 11 | 300 | 14.5 | 0.048 | | 620 | | 0.472 | | 199 |  |  |
| 12 | 330 | 15.5 | 0.046 | | 556 | | 0.423 | | 195.6 |  |  |
| 13 | 360 | 16.5 | 0.045 | | 635 | | 0.483 | | 195.5 |  |  |
| 14 | 390 | 17.5 | 0.044 | | 564 | | 0.429 | | 194.5 |  |  |
| 15 | 420 | 19 | 0.045 | | 565 | | 0.43 | | 194.4 |  |  |
| 16 | 450 | 21 | 0.046 | | 520 | | 0.396 | | 194.4 |  |  |
| 17 | 480 | 22 | 0.045 | | 494 | | 0.376 | | 194.7 |  |  |
| 18 | 510 | 24 | 0.047 | | 514 | | 0.391 | | 194.8 |  |  |
|  |  |  | |  | |  |  |  | |  |  |
|  |  |  | |  | |  |  |  | |  |  |

Table 1.2: Preliminary experimental trial for algal biomass sample of concentration at 0.55 g L^-1^ and flocculant addition of 0.15 g/L chitosan (normal sample with chitosan as flocculant)

| **Sl. no.** | **Time (min)** | **Height (cm)** | **Settling velocity (cm/min)** | **Cell number (10^4 cells/mL)** | **Optical density (at 680 nm)** | **Conductivity (µS/cm)** |
| --- | --- | --- | --- | --- | --- | --- |
| 1 | 0 | 0 | - | 879 | 0.669 | 231 |
| 2 | 15 | 1.5 | 0.100 | 863 | 0.657 | 232 |
| 3 | 30 | 5 | 0.166 | 829 | 0.631 | 235 |
| 4 | 45 | 9 | 0.200 | 815 | 0.62 | 230 |
| 5 | 60 | 12 | 0.200 | 813 | 0.619 | 233 |
| 6 | 75 | 16.5 | 0.220 | 838 | 0.638 | 233 |
| 7 | 90 | 18 | 0.200 | 792 | 0.603 | 233 |
| 8 | 105 | 20 | 0.190 | 830 | 0.632 | 233 |
| 9 | 120 | 21.5 | 0.179 | 841 | 0.64 | 234 |
| 10 | 135 | 23 | 0.170 | 817 | 0.622 | 234 |
| 11 | 150 | 24 | 0.160 | 815 | 0.62 | 234 |

Table 1.3: Preliminary experimental trial for algal biomass sample of concentration at 0.55 g L^-1^ and flocculant addition of 0.15 g/L bentonite (normal sample with bentonite as flocculant)

| **Sl. no.** | **Time (min)** | **Height (cm)** | **Settling velocity (cm/min)** | **Cell number (10^4 cells/mL)** | **Optical density (at 680 nm)** | **Conductivity (µS/cm)** |
| --- | --- | --- | --- | --- | --- | --- |
| 1 | 0 | 0 | - | 800 | 0.609 | 203 |
| 2 | 15 | 4 | 0.266 | 763 | 0.581 | 203 |
| 3 | 30 | 7.5 | 0.250 | 737 | 0.561 | 203 |
| 4 | 45 | 10 | 0.222 | 719 | 0.547 | 203 |
| 5 | 60 | 13 | 0.216 | 769 | 0.585 | 204 |
| 6 | 75 | 15.5 | 0.206 | 715 | 0.544 | 205 |
| 7 | 90 | 17.5 | 0.194 | 695 | 0.529 | 206 |
| 8 | 105 | 19 | 0.180 | 661 | 0.503 | 203 |
| 9 | 120 | 21 | 0.175 | 690 | 0.525 | 204 |
| 10 | 135 | 22.5 | 0.166 | 665 | 0.506 | 204 |
| 11 | 150 | 24 | 0.160 | 665 | 0.506 | 199 |

Table 1.4: Preliminary experimental trial for algal biomass sample of concentration at 0.55 g L^-1^ with flocculant addition of 0.15 g/L chitosan and 0.15 g/L bentonite (normal sample with flocculant combination as flocculant)

| **Sl. no.** | **Time (min)** | **Height (cm)** | **Settling velocity (cm/min)** | **Cell number (10^4 cells/mL)** | **Optical density (at 680 nm)** | **Conductivity (µS/cm)** |
| --- | --- | --- | --- | --- | --- | --- |
| 1 | 0 | 0 | - | 782 | 0.595 | 226 |
| 2 | 15 | 3.5 | 0.233 | 773 | 0.588 | 226 |
| 3 | 30 | 7 | 0.233 | 669 | 0.509 | 223 |
| 4 | 45 | 11 | 0.244 | 733 | 0.558 | 223 |
| 5 | 60 | 15.5 | 0.258 | 702 | 0.534 | 225 |
| 6 | 75 | 18 | 0.240 | 704 | 0.536 | 224 |
| 7 | 90 | 20 | 0.222 | 682 | 0.519 | 224 |
| 8 | 105 | 22 | 0.209 | 686 | 0.522 | 224 |
| 9 | 120 | 24 | 0.200 | 679 | 0.517 | 225 |
