# Supplementary material for: Batch Sedimentation Studies for Freshwater Green Alga Scenedesmus abundans Using Combination of Flocculants
Source: Front Chem. 2017 Jun 19;5:37. doi: 10.3389/fchem.2017.00037 (PMC5475385; doi:10.3389/fchem.2017.00037)
Supplement: Supplementary file 2 [file Table2.docx]

**Table A1**: Algae broth composition

| **Constituent** | **Composition (g/L)** |
| --- | --- |
| NaNO_3_ | 1 |
| K_2_HPO_4_ | 0.250 |
| MgSO_4_.7H_2_O | 0.513 |
| NH_4_Cl | 0.050 |
| CaCl_2_.2H_2_O | 0.058 |
| FeCl_2_.6H_2_O | 0.003 |
| Final pH | 7.0 +0.2 |

**Table A2:** Experimental matrix (with response values calculated from measurable values)

| Run | Concentration of algal biomass+ 0.05 (g/L) | Temperature+5 (deg C) | pH+0.1 | Concentration of flocculant+0.001 (g/L) | Flocculation efficiency+7.81 (% efficiency per hour) | Settling velocity+0.6 (cm/h) |
| --- | --- | --- | --- | --- | --- | --- |
| 1 | 1 | 32.5 | 4 | 0.005 | 11.64 | 13.44 |
| 2 | 1 | 50 | 12 | 0.005 | 96 | 102 |
| 3 | 0.55 | 15 | 12 | 0.3 | 52.38 | 29.88 |
| 4 | 0.55 | 50 | 9 | 0.1525 | 15.62 | 17.94 |
| 5 | 1 | 50 | 4 | 0.3 | 14.58 | 16.8 |
| 6 | 0.55 | 50 | 9 | 0.1525 | 20.88 | 18 |
| 7 | 0.1 | 15 | 4 | 0.005 | 36.15 | 14.94 |
| 8 | 0.1 | 32.5 | 12 | 0.005 | 63.63 | 28.5 |
| 9 | 1 | 32.5 | 9 | 0.3 | 16.28 | 14.82 |
| 10 | 0.1 | 15 | 9 | 0.005 | 22.5 | 14.16 |
| 11 | 0.55 | 15 | 12 | 0.3 | 28.69 | 27.78 |
| 12 | 0.55 | 32.5 | 4 | 0.1525 | 15.92 | 13.98 |
| 13 | 0.55 | 15 | 9 | 0.1525 | 17.03 | 15.78 |
| 14 | 0.55 | 32.5 | 9 | 0.005 | 21.72 | 12.18 |
| 15 | 1 | 15 | 4 | 0.3 | 23.8 | 16.5 |
| 16 | 0.1 | 15 | 9 | 0.3 | 28.57 | 13.86 |
| 17 | 1 | 15 | 12 | 0.1525 | 52.63 | 31.62 |
| 18 | 1 | 32.5 | 9 | 0.3 | 14.28 | 16.02 |
| 19 | 0.1 | 32.5 | 4 | 0.3 | 31.74 | 17.4 |
| 20 | 0.1 | 50 | 12 | 0.3 | 44.44 | 78.18 |
| 21 | 0.55 | 32.5 | 4 | 0.1525 | 22.12 | 13.86 |
| 22 | 0.55 | 32.5 | 9 | 0.005 | 18.31 | 12.24 |
| 23 | 1 | 15 | 9 | 0.005 | 32.47 | 19.62 |
| 24 | 0.1 | 15 | 12 | 0.1525 | 52.36 | 21.72 |
| 25 | 0.1 | 50 | 4 | 0.005 | 25.56 | 16.02 |

**Table A3**: Analysis of variance table [ANOVA for flocculation efficiency % per hour as response]

| **Source** | **Sum of**  **Squares** | **Degree of freedom** | **Mean**  **Square** | **F**  **Value** | **p-value**  **Prob > F** |  |
| --- | --- | --- | --- | --- | --- | --- |
| Model | 8419.94 | 10 | 841.99 | 13.82 | < 0.0001 | Significant |
| A-Concentration of algal biomass | 18.28 | 1 | 18.28 | 0.30 | 0.5925 |  |
| B-Temperature | 0.71 | 1 | 0.71 | 0.012 | 0.9153 |  |
| C-pH | 4228.57 | 1 | 4228.57 | 69.38 | < 0.0001 |  |
| D-Concentration of flocculant | 298.43 | 1 | 298.43 | 4.90 | 0.0440 |  |
| AC | 603.90 | 1 | 603.90 | 9.91 | 0.0071 |  |
| BC | 323.93 | 1 | 323.93 | 5.32 | 0.0370 |  |
| CD | 744.39 | 1 | 744.39 | 12.21 | 0.0036 |  |
| B^2 | 127.09 | 1 | 127.09 | 2.09 | 0.1707 |  |
| C^2 | 3416.68 | 1 | 3416.68 | 56.06 | < 0.0001 |  |
| D^2 | 93.34 | 1 | 93.34 | 1.53 | 0.2362 |  |
| Residual | 853.24 | 14 | 60.95 |  |  |  |
| Lack of Fit | 531.76 | 9 | 59.08 | 0.92 | 0.5715 | not significant |
| Pure Error | 321.48 | 5 | 64.30 |  |  |  |
| Cor Total | 9273.18 | 24 |  |  |  |  |

**Table A4**: Analysis of variance table [ANOVA for settling velocity (cm/h) as response]

| **Source** | **Sum of**  **Squares** | **df** | **Mean**  **Square** | **F**  **Value** | **p-value**  **Prob > F** |  |
| --- | --- | --- | --- | --- | --- | --- |
| Model | 10545.70 | 17 | 620.34 | 1411.28 | < 0.0001 | significant |
| A-Concentration of algal biomass | 65.14 | 1 | 65.14 | 148.19 | < 0.0001 |  |
| B-Temperature | 12.27 | 1 | 12.27 | 27.92 | 0.0011 |  |
| C-pH | 1.62 | 1 | 1.62 | 3.69 | 0.0961 |  |
| D-Concentration of flocculant | 8.72 | 1 | 8.72 | 19.84 | 0.0030 |  |
| AB | 10.23 | 1 | 10.23 | 23.27 | 0.0019 |  |
| AC | 87.37 | 1 | 87.37 | 198.76 | < 0.0001 |  |
| BC | 923.07 | 1 | 923.07 | 2100.02 | < 0.0001 |  |
| BD | 13.91 | 1 | 13.91 | 31.65 | 0.0008 |  |
| CD | 27.86 | 1 | 27.86 | 63.39 | < 0.0001 |  |
| A^2 | 7.10 | 1 | 7.10 | 16.16 | 0.0051 |  |
| B^2 | 172.06 | 1 | 172.06 | 391.44 | < 0.0001 |  |
| C^2 | 57.28 | 1 | 57.28 | 130.31 | < 0.0001 |  |
| D^2 | 82.12 | 1 | 82.12 | 186.82 | < 0.0001 |  |
| B^2C | 193.71 | 1 | 193.71 | 440.69 | < 0.0001 |  |
| BC^2 | 0.64 | 1 | 0.64 | 1.47 | 0.2653 |  |
| BD^2 | 54.35 | 1 | 54.35 | 123.64 | < 0.0001 |  |
| CD^2 | 28.24 | 1 | 28.24 | 64.25 | < 0.0001 |  |
| Residual | 3.08 | 7 | 0.44 |  |  |  |
| Lack of Fit | 0.14 | 2 | 0.071 | 0.12 | 0.8893 | not significant |
| Pure Error | 2.94 | 5 | 0.59 |  |  |  |
| Cor Total | 10548.78 | 24 |  |  |  |  |
