# Supplementary material for: Batch Sedimentation Studies for Freshwater Green Alga Scenedesmus abundans Using Combination of Flocculants
Source: Front Chem. 2017 Jun 19;5:37. doi: 10.3389/fchem.2017.00037 (PMC5475385; doi:10.3389/fchem.2017.00037)
Supplement: Supplementary file 3 [file Image1.PDF]

Supplementary Material

Batch sedimentation studies for freshwater green alga *Scenedesmus abundans* using combination of flocculants

Raghu Krishna Moorthy[1*], M. Premalatha[1], Muthu Arumugam[2]

[1]Department of Energy & Environment, National Institute of Technology, Tiruchirappalli, India
[2]Biotechnology Division, CSIR-National Institute of Interdisciplinary Science and Technology, Trivandrum, India

*** Correspondence:**
Raghu Krishna Moorthy
raghukmoorthy@gmail.com

1 FIGURES

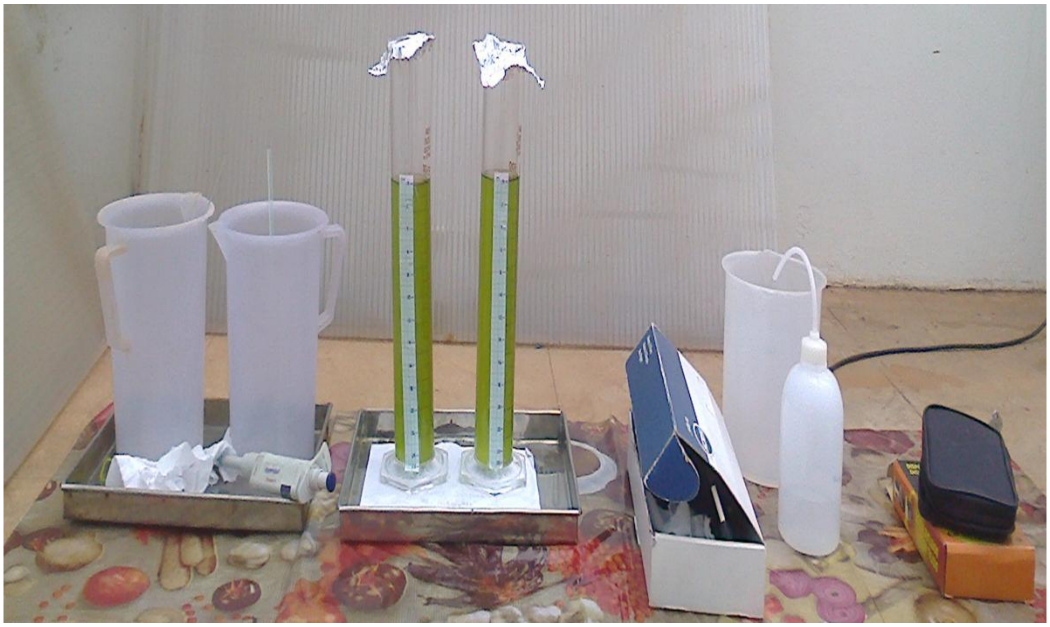

Fig. 1.1: Batch sedimentation studies for an algal solution of biomass concentration of 0.55 g/L at a pH of 9.4 (at algal cultivation shed for highly variable light intensity of 100 to 40000 lux and hot temperature condition varying between 34.1 ^{o}C and 46.1^{o}C on 28 September 2015)

Initial condition : time t = 0 **Final condition : time t = 8 hours**

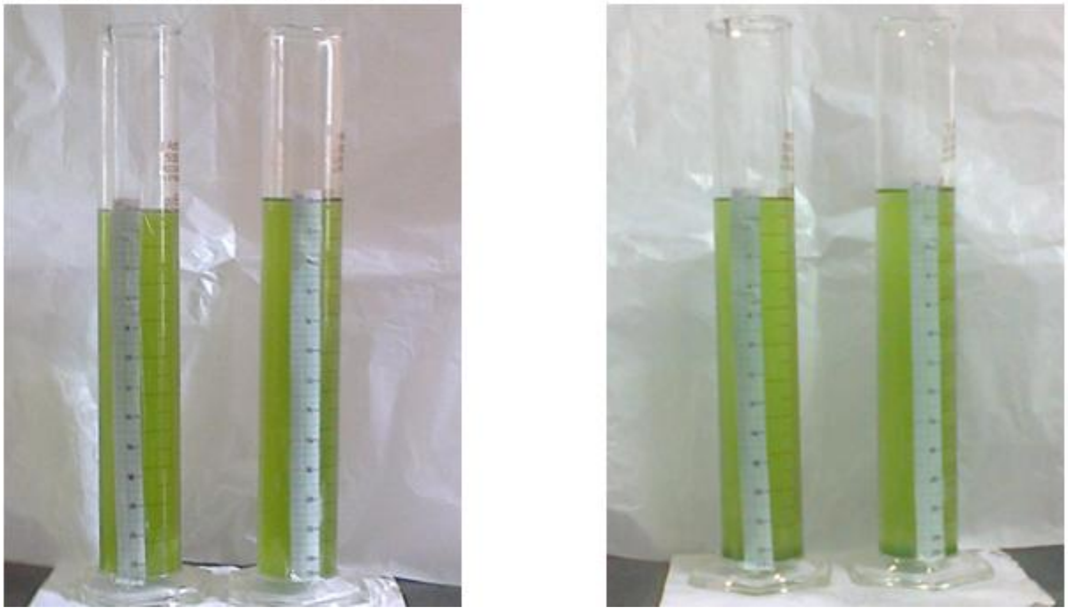

Fig. 1.2: Batch sedimentation studies (normal condition without any flocculant) for an algal solution of biomass concentration of 0.55 g/L at an initial pH of 9.8 and temperature of 33°C

Initial condition : time t = 0 **Final condition : time t = 1 hour**

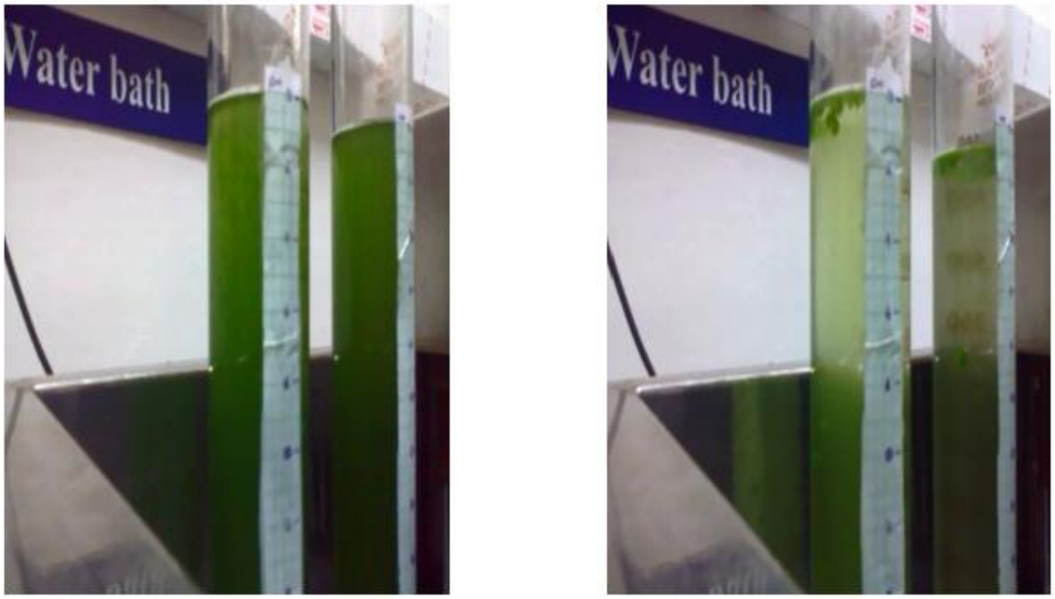

Fig. 1.3: Batch sedimentation studies (**optimal condition**) for an algal solution of biomass concentration of 1 g/L at an initial pH of 12 and temperature of 50°C with the addition of combination of flocculants (extracted chitosan and natural bentonite clay powder each of concentration 0.005 g/L

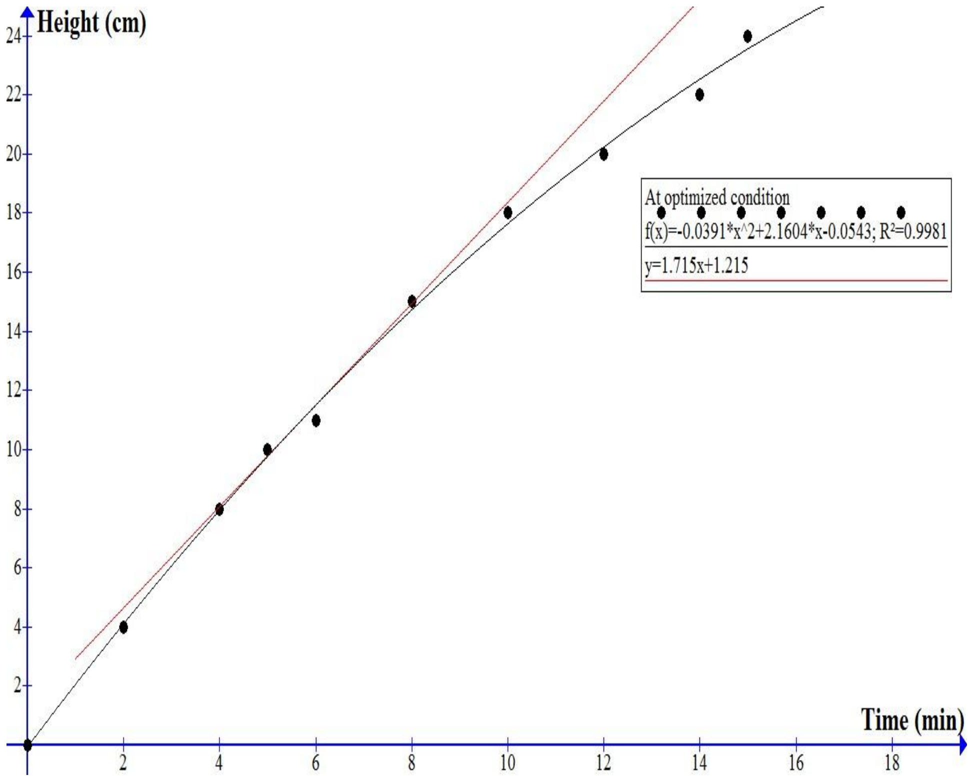

Fig. 1.4: Settling curve at optimized process condition for sedimentation-flocculation technique
